# Does coupling to ADP ribosylation factor 6 explain differences between muscarinic and other receptors in interaction with β-adrenoceptor-mediated smooth muscle relaxation?

**DOI:** 10.1007/s00210-022-02221-7

**Published:** 2022-02-17

**Authors:** Betül R. Erdogan, Martin C. Michel

**Affiliations:** 1 Department of Pharmacology, Faculty of Pharmacy, Izmir Katip Celebi University, Izmir, Turkey; 2 Department of Pharmacology, Johannes Gutenberg University, Langenbeckstr. 1, 55131 Mainz, Germany

**Keywords:** ADP ribosylation factor 6, β-Adrenoceptor, G-Protein-coupled receptor, Muscarinic receptor, Contraction

## Abstract

Numerous studies in airways, ileum, and urinary bladder have demonstrated that relaxation by β-adrenoceptor agonists has lower potency and/or efficacy when contraction was elicited by muscarinic receptor agonists as compared to other G-protein-coupled receptors, KCl, or basal tone, but the molecular mechanisms behind this relative resistance remain unclear. A paper by Huang et al. in this issue demonstrates that NAV2729, an inhibitor of ADP ribosylation factor 6, inhibits contraction of isolated blood vessels elicited by muscarinic receptor agonists, but not by α_1_-adrenoceptor agonists or KCl. Against this background, we discuss the role of ADP ribosylation factor 6 in cellular responses to G-protein-coupled receptor stimulation. While ADP ribosylation factor 6 apparently is the only promising molecular explanation for the relative resistance of smooth muscle contraction elicited by muscarinic agonists, the existing data are insufficient for a robust conclusion.

Muscarinic receptors are important mediators of smooth muscle contraction in various tissues including airways, gut, and urinary bladder; this largely involves the M_3_ subtype but M_2_ receptors can also affect smooth muscle tone (Hegde and Eglen 1999). M_3_ receptors typically couple to G-proteins of the G_q/11_ type leading to activation of a phospholipase C (PLC) (Caulfield and Birdsall 1998). Surprisingly, M_3_-mediated smooth muscle contraction is not explained by PLC activation, for instance in the bladder (Frazier et al. 2008), but alternative molecular mechanisms to elicit smooth muscle contraction have not been well-defined.

The potency and/or efficacy of β-adrenoceptor agonists to relax smooth muscle is lower when tested against muscarinic agonists such as carbachol than against agonists at other receptors, against passive tension or against receptor-independent contraction elicited by KCl (Dale et al. 2014). Such observations have been made in airways (Russel 1984; Raffestin et al. 1985; Ostrom and Ehlert 1999; Sarria et al. 2002; Naline et al. 2007), ileum (Ostrom and Ehlert 1999), and bladder (Longhurst and Levendusky 1999; Witte et al. 2011; Kanie et al. 2012) of multiple species including rat (Longhurst and Levendusky 1999; Michel and Sand 2009; Witte et al. 2011; Cernecka et al. 2014), guinea pig (Ostrom and Ehlert 1999), dog (Russel 1984), and human (Raffestin et al. 1985; Sarria et al. 2002; Naline et al. 2007; Kanie et al. 2012) and with agonists acting at histamine (Russel 1984; Raffestin et al. 1985; Ostrom and Ehlert 1999; Naline et al. 2007), 5-hydroxytryptamine (5-HT) (Michel and Sand 2009; Cernecka et al. 2014), bradykinin (Michel and Sand 2009; Cernecka et al. 2014), prostanoid receptors (Sarria et al. 2002), passive tone (Naline et al. 2007; Michel and Sand 2009; Cernecka et al. 2014), or KCl (Longhurst and Levendusky 1999; Michel and Sand 2009; Kanie et al. 2012). While most of the above studies have used general β-adrenoceptor agonists such as isoprenaline, similar findings have also been obtained with agonists selective for β_2_-adrenoceptors such as formoterol, indacaterol, salbutamol, and salmeterol in human bronchi (Naline et al. 2007) or fenoterol in rat bladder (Erdogan et al. 2021) or selective for β_3_-adrenoceptors such as TRK 380 in human bladder (Kanie et al. 2012) or KUC 7322 in rat bladder (Cernecka et al. 2014). However, it has remained elusive why smooth muscle contraction elicited by muscarinic receptor agonists is more resistant to relaxation by β-adrenoceptor agonists than that elicited by other stimuli. It appears logical that such selective resistance should be related to a signaling pathway activated preferentially by muscarinic receptors as compared to other receptors, but we are not aware of such signaling pathways.

A paper in this issue of the journal demonstrates that NAV2729, an inhibitor of ADP ribosylation factor 6 (ARF6), inhibits coronary vascular smooth muscle contraction elicited by the muscarinic agonists carbachol and methacholine but not that elicited by agonists at other receptors including α_1_-adrenoceptors, 5-HT, endothelin-1, or prostanoid TP receptors or those elicited by KCl (Huang et al. 2022). The same group had previously shown the existence of ARF6 expression human prostate smooth muscle tissue (Hennenberg et al. 2013) and that NAV2729 inhibited contraction in isolated human prostate strips by α_1_-adrenoceptor agonists, whereas that elicited by a prostanoid TP receptor agonist, endothelin-1 or by KCl, was not inhibited (Yu et al. 2019). Furthermore, NAV2729 inhibited contraction in human prostate smooth muscle cells, and this was also observed in ARF6 knockout cells (Wang et al. 2021). Inhibition of ARF6 by NAV2729 inhibited contraction elicited by α_1_-adrenoceptor agonists in human prostate (Yu et al. 2019) but not porcine blood vessels (Huang et al. 2022). Interestingly, the inhibition of contraction in human prostate was accompanied by an inhibition of ARF6 in pull-down assays, while ARF6 was not activated by noradrenaline, phenylephrine, or methoxamine (Yu et al. 2019). The selective inhibition of muscarinic receptor responses in the blood vessels (Huang et al. 2022) raises the possibility that coupling to ARF6 may be a mechanism that distinguishes inhibition by β-adrenoceptor agonists of responses to a muscarinic agonist as compared to those elicited by other means.

ARF6 is a small GTP-binding protein that contributes to several cellular processes including G-protein-coupled receptor (GPCR) trafficking, actin organization, and contractile response through diverse downstream component interaction (Fig. 1, Table 1). ARF6 function is modulated by two distinct components. Guanine nucleotide exchange factors (GEFs) mediate the activation of ARF6, whereas GTPase-activating proteins (GAPs) mediate inhibition. NAV2729 interferes in the formation of ARF6-GEF complex to inhibit the ARF6 activation (Yamauchi et al. 2017).

**Fig. 1:**
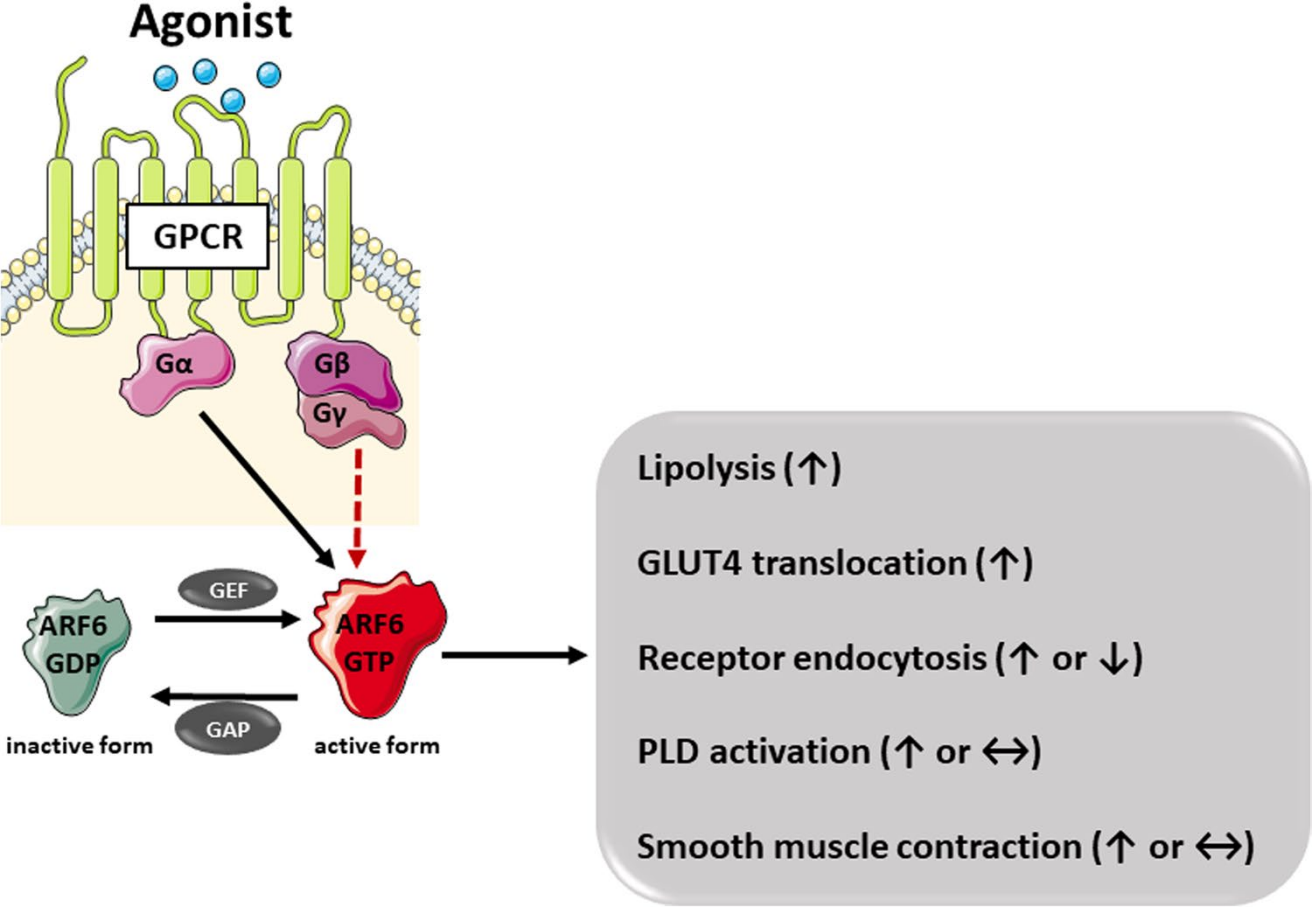
Schematic drawing of ARF6-mediated cellular effects in GPCRs agonist stimulation. Dashed red arrow, negative regulatory effect; black arrow, positive regulatory effect; GAP, GTPase-activating proteins; GDP, guanosine diphosphate; GEF, guanine nucleotide exchange factors; GTP, guanosine triphosphate; ↑, increase; ↓, decrease; ↔ , no effect

**Table 1:** ARF6-mediated responses through G-protein isoform/G-protein-coupled receptors

Reference	Cell line/tissue preparation	G-protein isoform/G-protein-coupled receptor	Main finding	ARF6-mediated effect
Bose et al. (2001)	3T3-L1 adipocytes	Gα11	Endothelin 1–induced GLUT4 translocation	↑
Bouschet et al. (2007)	HEK cells	Ca sensing receptor	Plasma membrane ruffling	↑
Chakraborti et al. (2017)	Human pulmonary artery smooth muscle cells	Prostanoid TP receptor	PLD2 and NADPH oxidase activation	↑
Chakraborti et al. (2018)	Human pulmonary artery smooth muscle cells	Endothelin receptor	PLD and NADPH oxidase activation	↑
Claing et al. (2001)	HEK293 cells	β_2_-adrenoceptor	Agonist-induced receptor endocytosis	↑
Cotton et al. (2007)	HEK293 cells	Angiotensin type 1 receptor	Agonist-induced membrane ruffling and cell migration	↑
Daher et al. (2008)	Endothelial cells	Endothelin ET_B_ receptor	Agonist-induced cell migration and angiogenesis	↑
Davies et al. (2014)	3T3-L1 adipocytes	Endothelin ET_A_ receptor	Agonist-induced lipolysis	↑
Delaney et al. (2002)	HeLa cells	M_2_ muscarinic receptor	Agonist-induced receptor endocytosis	↓
Hennenberg et al. (2013)	Human prostate smooth muscle tissue	α_1_-adrenoceptor	Receptor desensitization	↑
Herlemann et al. (2018)	Human prostate smooth muscle tissue	α-adrenoceptor Prostanoid TP receptor Endothelin receptor	Smooth muscle contraction	↑ ↑ ↑
Houndolo et al. (2005)	HEK293 cells	M_2_ muscarinic receptor Angiotensin type 1 receptor Vasopressin V_2_ receptor Endothelin type B receptor VIP receptor	Agonist-induced receptor endocytosis	↑ ↑ ↑ ↑ No effect
Huang et al. (2022)	Pig interlobar (ila) and coronary (ca) artery smooth muscle	Muscarinic receptor α_1_-Adrenoceptor 5-HT receptor Endothelin receptor Prostanoid TP receptor	Smooth muscle contraction	↑ (ca), no effect (ila) No effect (ca and ila) No effect (ca and ila) No effect (ca and ila) No effect (ca and ila)
Johnson et al. (2006)	COS7 cells	N376D mutant 5-HT_2A_ receptor WT 5-HT_2A_ receptor Purinergic P_2u_ receptor Thrombin PAR receptor Gonadotropin-releasing hormone receptor	PLD activation	↑ No effect ↑ ↑ ↑
Kanamarlapudi et al. (2012)	HEK 293 cells	Luteinizing hormone chorionic gonadotropin receptor	Receptor internalization	↑
Lawrence et al. (2005)	HEK293 cells	β_2_-Adrenoceptor	Receptor internalization	↑
Lawrence and Birnbaum (2001)	3T3-L1 adipocytes	G_αq_	Endothelin 1–induced GLUT4 translocation	↑
Le Stunff et al. (2000)	Female Wistar rat myometrium	G_βγ_	PLD activation	↓
Liu et al. (2010)	3T3-L1 adipocytes	β-Adrenoceptor	Agonist-induced lipolysis and endocytosis	↑
Macia et al. (2012)	HEK293 cells	β_2_-Adrenoceptor	Recycling of receptor	↓
Madziva and Birnbaumer (2006)	HEK 293-T cells	Vasopressin V_2_ receptor	Agonist-induced receptor endocytosis	No effect
Mitchell et al. (2003)	COS7 cells	M_3_ muscarinic receptors Purinergic P_2U_ receptor N376D mutant 5-HT_2A_ receptor	PLD1/2 activation PLD2 activation PLD2 activation	↑ ↑ No effect
Rankovic et al. (2009)	HEK293 cells	μ-Opioid receptor	Agonist-induced receptor endocytosis PLD2 activation	↑ ↑
Reiner and Nathanson (2008)	JEG-3 human choriocarcinoma cells	M_2_ muscarinic receptor M_4_ muscarinic receptor	Agonist-induced receptor endocytosis	↓ No effect
Yu et al. (2019)	Human prostate smooth muscle tissue	α_1_-adrenoceptor Prostanoid TP receptor Endothelin receptor	Smooth muscle contraction	↑ No effect No effect

ARF6 was shown to be a prerequisite component further promoting either clathrin- or caveolin-mediated pathway in agonist-induced endocytosis of several GPCRs such as β-adrenoceptor in adipocytes (Liu et al. 2010) and β_2_-adrenoceptor in HEK293 cells (Claing et al. 2001; Lawrence et al. 2005; Macia et al. 2012); M_2_ muscarinic receptors in HeLa cells (Delaney et al. 2002), in JEG-3 human choriocarcinoma cells (Reiner and Nathanson 2008), and in HEK293 cells (Houndolo et al. 2005); the luteinizing hormone chorionic gonadotropin receptor in HEK 293 cells (Kanamarlapudi et al. 2012); angiotensin type 1 receptor (Houndolo et al. 2005; Cotton et al. 2007); μ-opioid receptor (Rankovic et al. 2009); the vasopressin V_2_ receptor; and endothelin type B receptor (Houndolo et al. 2005) in HEK293 cells. Upon agonist stimulation, GPCR endocytosis was found mostly activated via ARF6-dependent pathway with some exceptions which show inhibitor regulatory effect of ARF6 in M_2_ muscarinic receptor (Delaney et al. 2002; Reiner and Nathanson 2008) and in β_2_-adrenoceptor (Macia et al. 2012) internalization. Furthermore, VIP receptor internalization was not affected by ARF6 depletion (Houndolo et al. 2005). ARF6 involvement in trafficking did not exist for some receptors such as M_4_ muscarinic receptor (Reiner and Nathanson 2008) and vasopressin V_2_ receptor (Madziva and Birnbaumer 2006). ARF6 requirement in endocytosis was mostly demonstrated in agonist-induced settings, which may not reflect the ARF6 function for basal condition for the same receptor (Cotton et al. 2007). Moreover, ARF6 involvement of muscarinic receptor internalization has mostly been studied with the M_2_ subtype because of the well-defined, clathrin-dependent pathway-mediated internalization of M_1_, M_3_, and M_4_ receptors (Reiner and Nathanson 2008).

Phospholipase D (PLD) is known to be involved in smooth muscle contraction through PKC activation but its contribution in urinary bladder contraction was proposed to be minor (Frazier et al. 2008). ARF6-mediated PLD activation was reported by several researchers in in vivo animal (Le Stunff et al. 2000) and in vitro cultured cell line studies (Mitchell et al. 2003; Johnson et al. 2006; Rankovic et al. 2009; Chakraborti et al. 2017; Charles et al. 2018). In human pulmonary artery smooth muscle cells (HPASMCs), stimulation of prostanoid TP receptor stimulates cytohesin-1 coupling to ARF6 which further leads to PLD2 isoform and subsequent NADPH oxidase activation (Chakraborti et al. 2017). In HPASMCs, involvement of ARF6 in endothelin-1-induced PLD and NADPH oxidase activation was shown by same study group (Chakraborti et al. 2018). ARF6 was shown to be involved in PLD activation in N376D mutant 5-HT_2A_-stimulated PLD activation but not in mediated via WT 5-HT_2A_ stimulation (Johnson et al. 2006). This study also showed the sensitivity of other class A GPCRs which contain DPxxY motif such as purinergic P_2u_, thrombin PAR, and gonadotropin-releasing hormone receptor to ARF6 for further PLD activation in COS7 cells (Johnson et al. 2006). In the same cell line, agonist-stimulated M_3_ activation induced both PLD1/2 activation through ARF6-mediated pathway, whereas PLD2 activation was found linked to PKC and ARF6 in purinergic P_2U_ receptor and to only PKC in N376D mutant 5-HT_2a_ receptor (Mitchell et al. 2003). ARF6 is involved in the promotion of prostate smooth muscle contraction. Inhibition of ARF6 activation by cytohesin (a GEF) inhibitor resulted in reduced noradrenaline, phenylephrine-, thromboxane A_2_-, and endothelin-1- and endothelin-3-induced contraction (Herlemann et al. 2018). In vascular smooth muscle cell, both ARF1 and ARF6 are involved in actin polymerization which subsequently migrate and proliferate but only ARF1 affected contractile responses (Charles et al. 2018). However, in a latter study, ARF6 was found to promote contraction and proliferation in human prostate stromal cells (WPMY-1) (Wang et al. 2021).

ARF6 activation interrupts the recycling of β_2_-adrenoceptors and lead desensitization of receptor in HEK293 cells (Macia et al. 2012); similarly, it is postulated that ARF6 may involve in α_1_-adrenoceptor desensitization in human prostate tissue (Hennenberg et al. 2013). In contrast, ARF6-mediated endocytosis was suggested beneficial in development of opioid tolerance through preventing receptor desensitization HEK293 cells (Rankovic et al. 2009).

Other effects of ARF6 include the calcium-sensing receptor-mediated plasma membrane ruffling which is required for chemotaxis in HEK cells (Bouschet et al. 2007). In endothelial cells, endothelin ET_B_ receptor stimulation by endothelin 1 results in ARF6 activation which facilitates cell migration via actin reorganization. Moreover, endothelin 1 stimulation did not promote capillary tube formation in ARF6 depleted cells which indicates ARF6 involvement in angiogenesis (Daher et al. 2008). ARF6 also has regulatory effects on metabolic pathway. Depletion of ARF6 resulted in inhibition of isoproterenol-induced lipolysis in 3T3-L1 adipocytes. ARF6 mRNA and protein level was found higher in WAT tissue of *ob/ob* mice compared to obesity resistance mice (Liu et al. 2010). In 3T3-L1 adipocytes, endothelin ET_A_ receptor-mediated lipolysis was found to be dependent downstream ARF6-ERK1/2 signaling (Davies et al. 2014). Additionally, endothelin 1 stimulated GLUT4 translocation through heterotrimetric G_q_ protein signaling pathway activated by ARF6 in 3T3-L1 adipocytes (Bose et al. 2001; Lawrence and Birnbaum 2001) and specifically G_α11_ isoform of G_αq_ family.

Taken together, the above data demonstrate that ARF6 is involved in cellular processes and is activated by cellular effects stimulated by various GPCR, including smooth muscle contraction. Within a given tissue, the involvement of ARF6 in pathways leading to smooth muscle contraction, e.g., in arteries or prostate, appears to be preferential for some GPCR (muscarinic receptors and α_1_-adrenoceptors, respectively) over others apparently coupling to the same G-proteins. Some of these data are in line with the hypothesis that coupling to ARF6 may explain the relative resistance of smooth muscle contraction elicited by muscarinic receptors as compared to other GPCR or receptor-independent contraction such as receptor desensitization to relaxation by β-adrenoceptor agonists. However, not all data support this hypothesis. Thus, the role of ARF6 in this phenomenon cannot be considered proven, but it remains as a reasonable molecular candidate to explain the resistance of muscarinic receptor-mediated smooth muscle contraction against relaxation. Further studies at the molecular level are required to further explore this, specifically studies in which the role of agonists at various GPCR is compared quantitatively.
